# Seizure outcomes following epilepsy surgery in pediatric and young adult patients with high‐grade brain tumors: Results from a European survey

**DOI:** 10.1111/epi.18323

**Published:** 2025-03-01

**Authors:** Robert Lersch, Till Hartlieb, Tom Pieper, Manfred Kudernatsch, Wiebke Hofer, Carmen Barba, Renzo Guerrini, Flavio Giordano, Marianna Pommella, Susanne Schubert‐Bast, Steffen Syrbe, Ricardo Rego, Jorge Pinheiro, Martha Feucht, Alexander Beck, Roland Coras, Ingmar Blumcke, Michael Alber, Moritz Tacke, Jan Rémi, Christian Vollmar, Mathias Kunz, Jay Shetty, Ailsa McLellan, Drahoslav Sokol, Jothy Kandasamy, Kerstin Alexandra Klotz, Victoria San Antonio‐Arce, Andreas Schulze‐Bonhage, Joshua Pepper, William B. Lo, Alexis Arzimanoglou, Stefano Francione, Christian J. Braun, Ingo Borggraefe

**Affiliations:** ^1^ Department of Pediatrics, Dr. von Hauner Children's Hospital Ludwig Maximilian University Hospital Munich Germany; ^2^ Center for Pediatric Neurology, Neurorehabilitation, and Epileptology Schoen Clinic Vogtareuth Germany; ^3^ Rehabilitation, Transition, and Palliation Paracelsus Medical University Salzburg Salzburg Austria; ^4^ Center for Neurosurgery, Epilepsy Surgery, Spine Surgery, and Scoliosis Surgery Schoen Clinic Vogtareuth Vogtareuth Germany; ^5^ Neuroscience and Medical Genetics Department Meyer Children's Hospital Istituto di Ricovero e Cura a Carattere Scientifico, member of European Reference Network EpiCARE Florence Italy; ^6^ Department of Neuroscience, Psychology, Drug, and Child's Health (NEUROFARBA) University of Florence Florence Italy; ^7^ Goethe University Frankfurt Center of Neurology and Neurosurgery, Epilepsy Center Frankfurt Rhine‐Main Frankfurt am Main Germany; ^8^ LOEWE Center for Personalized and Translational Epilepsy Research Goethe University Frankfurt Frankfurt am Main Germany; ^9^ Goethe University Frankfurt, University Hospital for Children and Adolescents Pediatric Epileptology Frankfurt am Main Germany; ^10^ Center for Pediatrics and Adolescent Medicine, Division of Pediatric Epileptology Heidelberg University Heidelberg Germany; ^11^ Neurophysiology Unit, Neurology Department Centro Hospitalar Universitário de São João Porto Portugal; ^12^ Department of Anatomic Pathology Centro Hospitalar Universitário de São João Porto Portugal; ^13^ Department of Pediatric and Adolescent Medicine Medical University of Vienna, member of European Reference Network EpiCARE Vienna Austria; ^14^ Center for Neuropathology and Prion Research Faculty of Medicine, LMU Munich Munich Germany; ^15^ Department of Neuropathology University Hospital Erlangen, member of European Reference Network EpiCARE Erlangen Germany; ^16^ Department of Pediatric Neurology and Developmental Medicine University Children's Hospital Tübingen Germany; ^17^ Division of Pediatric Neurology and Developmental Medicine, Department of Pediatrics Dr. von Hauner Children's Hospital, Ludwig Maximilian University Hospital and Epilepsy Center Munich Munich Germany; ^18^ Department of Neurology University Hospital, Ludwig Maximilian University Munich Munich Germany; ^19^ Comprehensive Epilepsy Center University Hospital, Ludwig Maximilian University Munich Munich Germany; ^20^ Department of Neurosurgery University Hospital, Ludwig Maximilian University Munich Munich Germany; ^21^ Department of Child Life and Health, Institute for Regeneration and Repair University of Edinburgh Edinburgh UK; ^22^ Department of Paediatric Neurosciences Royal Hospital for Children and Young People Edinburgh UK; ^23^ Department of Neuropediatrics University Hospital Bonn, member of European Reference Network EpiCARE Bonn Germany; ^24^ Epilepsy Center University Medical Center, University of Freiburg, member of European Reference Network EpiCARE Freiburg Germany; ^25^ Department of Neurosurgery Birmingham Children's Hospital Birmingham UK; ^26^ Pediatric Epilepsy Department member of European Reference Network EpiCARE, University Hospitals of Lyon (HCL) Lyon France; ^27^ Neurology Department, Epilepsy Unit, European Reference Network EpiCARE coordination Hospital San Juan de Dios Barcelona Spain; ^28^ Department of Neuroscience Claudio Munari Epilepsy Surgery Center, member of European Reference Network EpiCARE Milan Italy

**Keywords:** children, drug‐resistant epilepsy, epilepsy surgery, high‐grade brain tumors, seizure freedom

## Abstract

**Objective:**

Epilepsy surgery is a standard treatment for drug‐resistant epilepsy, resulting in seizure freedom in a significant number of cases. Although frequently performed for low‐grade brain tumors, it is rarely considered for high‐grade tumors, despite the impact of chronic epilepsy on quality of life and cognition.

**Methods:**

This retrospective multicenter study across 43 European centers evaluated epilepsy surgery outcomes in children with high‐grade brain tumors (World Health Organization grades III and IV). Two cohorts of patients younger than 25 years were studied: (1) those undergoing epilepsy surgery after tumor resection (*n* = 14) and (2) those initially suspected of low‐grade lesions but diagnosed with high‐grade brain tumors postsurgery (*n* = 11).

**Results:**

Eighty percent of patients achieved seizure freedom 1 year after last epilepsy surgery: 71% in Cohort 1 and 91% in Cohort 2. Eighty‐four percent were free of disabling seizures (Engel IA–D) after a median follow‐up period of 4.3 years (range = 1–15.9 years). No surgery‐related deaths occurred. Thirty‐two percent of children experienced persistent morbidity, including motor dysfunction, visual impairment, persistent seizures, cognitive deficits, and hydrocephalus.

**Significance:**

Epilepsy surgery is effective for medically refractory epilepsy in children with high‐grade central nervous system tumors and should be considered early, as seizure freedom is achieved in the majority of patients. Despite involving numerous epilepsy centers, only 25 patients were recruited, indicating that this method is rarely considered for high‐grade brain tumor patients with medically refractory epilepsy.

## Graphical abstract

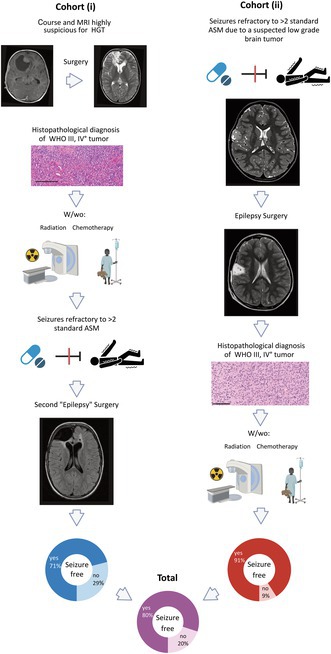

**Key points**

- Epilepsy surgery is effective for children with high‐grade brain tumors.
- Approximately three quarters of patients were seizure‐free after surgery, with no associated mortality.
- Seizure freedom rates comparable to other pediatric studies were achieved.

## 1 INTRODUCTION

Tumors of the central nervous system (CNS) are the most common solid tumors in children, accounting for approximately 20% of all neoplasms, and are the leading cause of cancer‐related death in this age group.^1^, ^2^ Survivors experience high postcancer morbidity, which is often linked to the tumor's location and the treatments used. Epilepsy affects approximately 25% of patients either at the onset or during the course of their disease.^3^, ^4^, ^5^, ^6^ The presence of epileptic seizures negatively impacts both cognition and quality of life, regardless of the administered cancer therapy.^5^ A substantial number of patients with epilepsy experience drug‐resistant seizures, defined as the failure of two well‐tolerated antiseizure medications (ASMs) in monotherapy or in combination.^7^, ^8^, ^9^ Early treatment of refractory epilepsy is essential to prevent cognitive and psychological consequences of chronic epilepsy.^10^

Surgery is an effective therapy for children with drug‐resistant focal epilepsy.^11^ Systematic reviews and meta‐analyses consistently demonstrate that epilepsy surgery is superior to ongoing ASM treatment in achieving seizure freedom and enhancing overall quality of life.^12^, ^13^, ^14^, ^15^ Furthermore, most patients show cognitive improvement following epilepsy surgery.^16^, ^17^ Surgical interventions include a range of procedures, such as lesionectomy, lobar or multilobar resection, hemispherotomy, or implantation of a vagus nerve stimulator.^11^, ^13^ Using neuroimaging techniques such as magnetic resonance imaging (MRI), nuclear imaging (fluorodeoxyglucose positron emission tomography, single photon emission computed tomography), and noninvasive and invasive electroencephalography (EEG; subdural grids), the aim of presurgical assessment is to identify the epileptogenic zone and to define eloquent areas to be spared during surgery.^11^, ^13^

Surgery is the standard of care for children with drug‐resistant seizures due to low‐grade brain tumors.^18^, ^19^ A large body of data demonstrates its efficacy in achieving seizure freedom in up to 80% of patients.^20^, ^21^ However, epilepsy surgery is very rarely considered for long‐term survivors of high‐grade CNS tumors. Consequently, data on the efficacy of this treatment in these patients are limited to small case series of four patients or fewer.^22^, ^23^ To address this, we conducted a retrospective study to evaluate the feasibility and potential benefits of epilepsy surgery in patients with high‐grade brain tumors, aiming to contribute to the ongoing discussion on its application in this context.

Here, we present retrospective data from a survey of 43 European epilepsy centers evaluating the outcome of epilepsy surgery in patients with high‐grade (World Health Organization [WHO] grades III and IV) pediatric brain tumors. We considered two different cohorts of patients: (1) those who underwent epilepsy surgery after tumor resection of high‐grade CNS tumors and (2) those who initially underwent epilepsy surgery under suspicion of a low‐grade lesion but received a final histopathologic diagnosis of high‐grade brain tumor.

## 2 MATERIALS AND METHODS

### 2.1 Study design

We conducted a retrospective multicenter study. Forty‐three centers all over Europe were contacted, mainly through the informal association of the European Taskforce for Childhood Epilepsy Surgery (U‐TASK), the European Reference Network for Rare and Complex Epilepsies (ERN EpiCARE), and ESNEK (survey of rare neurological diseases in childhood, Germany). Eleven centers were able to contribute patients to the study meeting the inclusion criteria. The data for this study were collected through a retrospective analysis using questionnaires sent to the participating epilepsy centers, which gathered relevant clinical and pathological information (Table S1). The study was approved by the local ethical board (# 22‐0602).

In accordance with the local ethics committee, informed consent from the patients was not required, as all data were pseudonymized. Patients or the public were not involved in the design, conduct, reporting, or dissemination plans of the study.

### 2.2 Inclusion criteria

Inclusion criteria of Cohort 1 comprised patients diagnosed with supratentorial high‐grade brain tumors (WHO grades III and IV) who developed medically refractory epilepsy after first tumor resection and received a second brain surgery for the treatment of epilepsy (epilepsy surgery) at age <25 years. Inclusion criteria of Cohort 2 comprised patients who underwent primary epilepsy surgery due to medically refractory epilepsy at <25 years with the initial radiological diagnosis of a WHO grade I or II tumor, in whom the pathological investigation of the specimen revealed features of a WHO grade III or IV tumor. Medically refractory epilepsy was defined as persistent seizure occurrence despite treatment with two first‐line antiepileptic drugs at adequate doses and with good tolerability.^7^, ^8^, ^9^ Patients with low‐grade and infratentorial tumors were excluded. The age limit was set at 25 years to include young adults, as they often share similar tumor biology, seizure characteristics, and treatment responses with adolescents and are frequently treated at pediatric epilepsy centers during the transition to adult care.

The histologic diagnoses were made according to the WHO classification at the time of diagnosis and were conducted within the neuropathology department of each respective center. In addition to histological criteria, tumor classification was based on immunohistochemistry, methylation profiling, and copy number variation in nine patients. In 15 cases, only immunohistochemistry was performed, with additional single‐gene analyses conducted in three of these patients. The original pathology report for one patient could not be obtained.

### 2.3 Endpoints

The primary endpoint was the number of patients gaining seizure freedom 1 year after surgery. Secondary endpoints were the distribution of seizure outcome according to the Engel classification system,^24^ and morbidity and mortality rates. Furthermore, the dataset includes demographic and clinical data pertaining to tumor type and therapy, epilepsy and therapy, cognition, survival, and morbidity.

### 2.4 Statistical analysis

We used R (version 4.4.1)^25^ for statistical analysis. We assessed the relationship between seizure outcomes and variables such as age at epilepsy onset, age at the time of surgery, and duration from epilepsy onset to surgery using the Mann–Whitney *U*‐test. The effects of presurgical seizure frequency and the type of surgical procedure on outcomes were analyzed with the chi‐squared test. To compare pre‐ and postsurgical intelligence quotient (IQ) outcomes, we employed the Mann–Whitney U test. A two‐tailed significance level of .05 was applied for all statistical analyses.

## 3 RESULTS

### 3.1 Demographic data and tumor pathology

A total of 25 patients were included in the study, comprising 17 males (68%) and eight females (32%). The median age at onset of epilepsy was 8 years (range = 0–16 years). Most patients (84%) had daily seizures before surgery (Table 1, Figure 2A), with a cumulative total number of ASMs of a maximum of 10 (median = 3, range = 1–10). In Cohort 2, two patients were treated with only one ASM in total before surgery.

**TABLE 1:** Demographic, presurgical, and postsurgical data.

Characteristic	Pooled cohort, *N* = 25	Cohort 1, *n* = 14	Cohort 2, *n* = 11
Patient gender	Male: *n* = 17; 68% Female: *n* = 8; 32%	Male: *n* = 11; 79% Female: *n* = 3; 21%	Male: *n* = 6; 55% Female: *n* = 5; 45%
Patient age at epilepsy manifestation, years	Median: 8 Range: 0–16	Median: 8 Range: 0–14	Median: 9 Range: 1–16
Patient age at last epilepsy surgery, years	Median: 11 Range: 1–22	Median: 11 Range: 3–22	Median: 10 Range: 1–18
Duration from epilepsy manifestation to last epilepsy surgery, years	Median: 2.5 Range: 0–20.2	Median: 2.7 Range: 1.5–20.2	Median: 1.1 Range: 0–7.5
Seizure frequency before epilepsy surgery	Daily: *n* = 21; 84% Weekly: *n* = 3; 12% Monthly: *n* = 1; 4%	Daily: *n* = 13; 93% Weekly: *n* = 1; 7%	Daily: *n* = 8; 73% Weekly: *n* = 2; 18% Monthly: *n* = 1; 9%
WHO tumor classification	III: *n* = 17; 68% IV: *n* = 8; 32%	III: *n* = 9; 64% IV: *n* = 5; 36%	III: *n* = 8; 73% IV: *n* = 3; 27%
Tumor pathology		Anaplastic glioma: *n* = 4 Anaplastic ependymoma: *n* = 2 ATRT: *n* = 2 Plexus carcinoma: *n* = 2 Glioblastoma: *n* = 2 Anaplastic pilocytic astrocytoma: *n* = 1 PNET: *n* = 1	Pleomorphic xanthoastrocytoma: *n* = 2 Anaplastic glioma: *n* = 2 Anaplastic ganglioglioma: *n* = 2 Anaplastic ependymoma: *n* = 1 Diffuse hemispheric glioma: *n* = 1 DNET: *n* = 1 DGONC: *n* = 1 ETMR: *n* = 1
Patient seizure‐free 1 year after last epilepsy surgery	Yes: *n* = 20; 80% No: *n* = 5; 20%	Yes: *n* = 10; 71% No: *n* = 4; 29%	Yes: *n* = 10; 91% No: *n* = 1; 9%
Postop seizure outcome after last epilepsy surgery (Engel classification)	Median follow‐up: 4.3 years Range: 1–15.9 years IA: *n* = 19; 76% IB: *n* = 1; 4% IC: *n* = 1; 4% IIA: *n* = 1; 4% IIB: *n* = 1; 4% IID: *n* = 2; 8%	Median follow‐up: 4.8 years Range: 1–15.9 years IA: *n* = 10; 71% IB: *n* = 1; 7% IIA: *n* = 1; 7% IIB: *n* = 1; 7% IID: *n* = 1; 7%	Median follow‐up: 2.5 years Range: 1–14.3 years IA: *n* = 9; 82% IC: *n* = 1; 9% IID: *n* = 1; 9%
Postsurgical sustained morbidity related to surgery	Yes: *n* = 8; 32% No: *n* = 17; 68%	Yes: *n* = 4; 29% No: *n* = 10; 71%	Yes: *n* = 4; 36% No: *n* = 7; 64%
Mortality associated with epilepsy surgery	Yes: *n* = 0; 0% No: *n* = 25; 100%	Yes: *n* = 0; 0% No: *n* = 14; 100%	Yes: *n* = 0; 0% No: *n* = 11; 100%
Mortality due to progressive tumor disease	Yes: *n* = 0; 0% No: *n* = 25; 100%	Yes: *n* = 0; 0% No: *n* = 14; 100%	Yes: *n* = 0; 0% No: *n* = 11; 100%

*Note*: Anaplastic glioma includes anaplastic astrocytoma, anaplastic oligodendroglioma, and anaplastic oligoastrocytoma.

Abbreviations: ATRT, atypical teratoid/rhabdoid tumor; DGONC, diffuse glioneuronal tumor with oligodendrogliomalike features and nuclear clusters; DNET, dysembryoplastic neuroepithelial tumor; ETMR, embryonal tumor with multilayered rosettes; PNET, primitive neuroectodermal tumor; WHO, World Health Organization.

**FIGURE 1:**
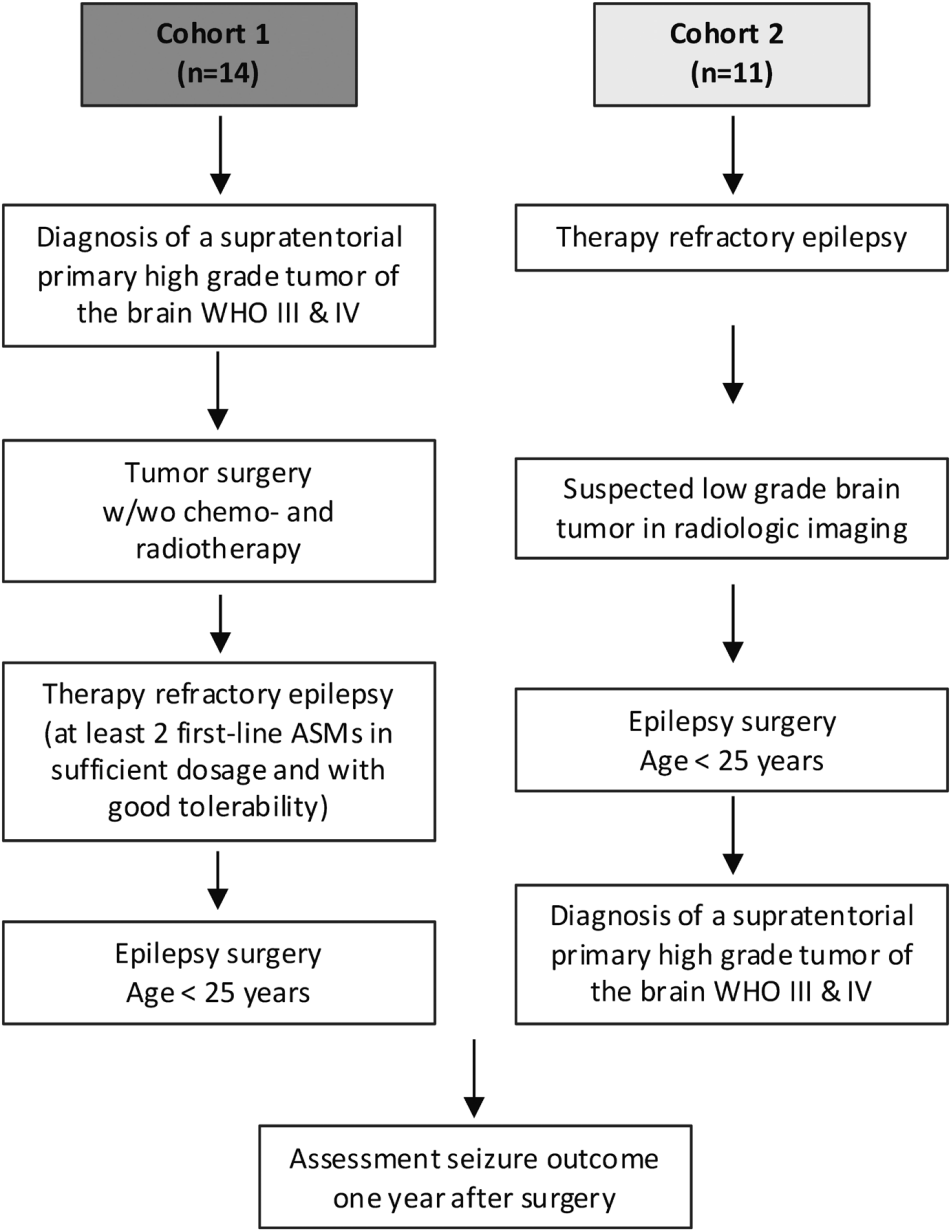
Enrollment criteria for both cohorts. ASM, antiseizure medication; w/wo, with/without; WHO, World Health Organization.

**FIGURE 2:**
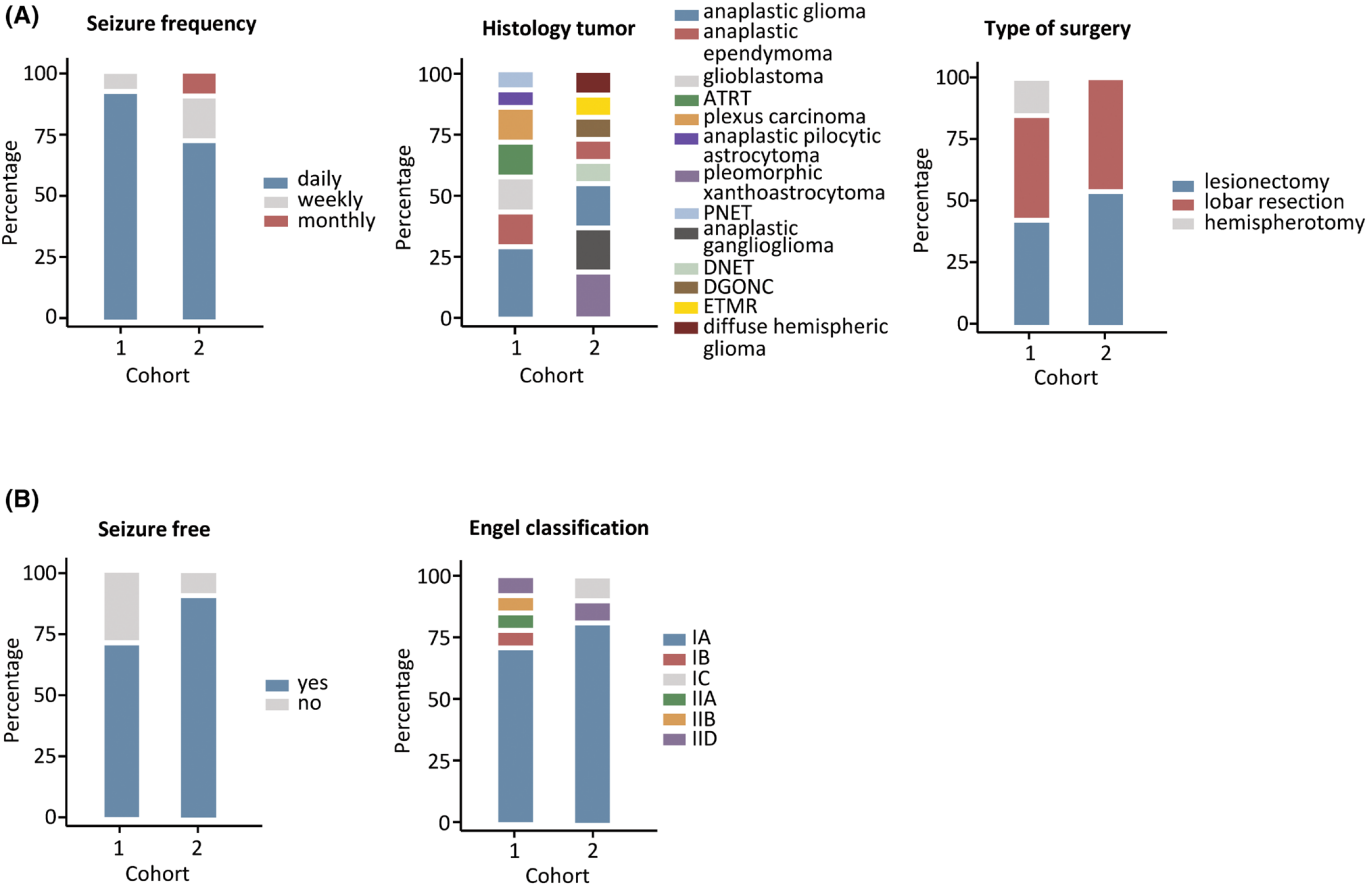
(A) Presurgical seizure frequency, tumor histology, and surgical procedure. (B) Postsurgical seizure outcome. (A) Bar graphs illustrating presurgical seizure frequency, tumor histology and the type of epilepsy surgery performed (last surgery). ATRT, atypical teratoid/rhabdoid tumor; DGONC, diffuse glioneuronal tumor with oligodendrogliomalike features and nuclear clusters; DNET, dysembryoplastic neuroepithelial tumor; ETMR, embryonal tumor with multilayered rosettes; PNET, primitive neuroectodermal tumor. Anaplastic glioma includes anaplastic astrocytoma, anaplastic oligodendroglioma, and anaplastic oligoastrocytoma. (B) Bar graphs depicting postsurgical seizure freedom 1 year after the last epilepsy surgery (if multiple surgeries were performed) and surgical outcomes according to the Engel classification.^24^

Cohort 1 included 14 patients with a history of high‐grade brain tumor that was surgically removed and who subsequently underwent epilepsy surgery for medically refractory epilepsy (Table 1, for patient disposition see Figure 1, left column, Table S2). The median age at tumor manifestation was 2 years (range = 0–6 years), and the median age at the time of the last epilepsy surgery was 11 years (range = 3–22 years). Epilepsy was diagnosed a median of 4 years after tumor surgery (range = −.3 to 13.3 years), with one patient with an anaplastic astrocytoma experiencing seizures 3 months before tumor surgery, coinciding with the time of tumor manifestation. The cohort included nine patients with WHO grade III and five with WHO grade IV tumors (Table 1). The histology of the tumors included four anaplastic gliomas (astrocytomas and oligodendrogliomas), two anaplastic ependymomas, two atypical teratoid rhabdoid tumors (ATRTs), two plexus carcinomas, two glioblastomas, one anaplastic pilocytic astrocytoma, and one primitive neuroectodermal tumor (Figure 2A). Postoperative treatment comprised radiation (*n* = 12) and chemotherapy (*n* = 14).

Cohort 2 included 11 patients who underwent primary epilepsy surgery due to medically refractory epilepsy (Table 1, for patient disposition see Figure 1, right column, Table S3). The median age at epilepsy surgery was 10 years (range = 1–18 years). The tumors were histologically classified as WHO grade III (73%) and IV (27%; Table 1). Histologic findings revealed the presence of two pleomorphic xanthoastrocytomas, two anaplastic gliomas, two anaplastic gangliogliomas, one anaplastic ependymoma, one diffuse hemispheric glioma, one anaplastic dysembryoplastic neuroepithelial tumor (DNET), one diffuse glioneuronal tumor with oligodendrogliomalike features and nuclear clusters, and one embryonal tumor with multilayered rosettes (Figure 2A). Malignant transformation of a DNET is rare,^26^, ^27^ and in this case, the diagnosis was based solely on immunohistochemistry (Table S3). Therefore, the possibility of misclassification cannot be entirely excluded. Further oncologic treatment included chemotherapy in seven patients and radiation in eight patients; one patient underwent subsequent tumor surgery after initial lobar resection.

### 3.2 Presurgical evaluation, type of surgery, and histopathological findings

The presurgical evaluation involved long‐term surface EEG monitoring in 22 patients (no data available for three patients) and invasive monitoring in five of 13 patients (no data available for 12 patients). Intraoperative electrocorticography was performed in 13 of 15 patients (no data available for 10 patients; see Tables S2 and S3).

In Cohort 1, the surgeries performed included lesionectomy (*n* = 8), lobar resection (*n* = 4), and hemispherotomy (*n* = 2) for the first epilepsy surgery (Figure 2A). Hemispherotomy was indicated in both patients due to extensive, multilobar lesions (Figure S1). Three patients required a second epilepsy surgery, with lobar resection performed in two patients and further complete disconnection after hemispherotomy in one patient. Histological analysis of the resected tissue identified reactive gliosis in seven patients. One patient with an initial diagnosis of ATRT showed meningioangiomatosis. Two patients with an initial diagnosis of ependymoma retained portions of their original tumors. In one patient initially diagnosed with an astrocytoma, histologic examination of the resected tissue revealed features consistent with an atypical ganglioglioma (WHO grade II). Similarly, histological analysis of the epilepsy surgery resection in a patient initially diagnosed with an anaplastic pilocytic astrocytoma (differential diagnosis at the time in the pathology report: anaplastic pleomorphic xanthoastrocytoma) revealed components of a pleomorphic xanthoastrocytoma, this time classified as WHO grade II.

In Cohort 2 surgery included lesionectomy in six patients and lobar resection in five patients (Figure 2A).

### 3.3 Seizure outcome

Eighty percent of patients were seizure‐free 1 year after the last epilepsy surgery. Eighty‐four percent were free of disabling seizures (Engel class IA–D) after a median follow‐up period of 4.3 years (range = 1–15.9 years; Table 1, Figure 2B).

In Cohort 1, 10 of 14 patients (71%) were seizure‐free 1 year after the last surgery. The distribution of epilepsy surgical outcome according to Engel after a median follow‐up period of 4.8 years (range = 1–15.9 years) was as follows: 10 patients were classified as IA and one each as IB, IIA, IIB, and IID, all of whom had an improved seizure outcome (Figure 2B), 79% being free of disabling seizures (Engel class IA–D; Table 1, Figure 2B).

In Cohort 2, 91% were seizure‐free 1 year after surgery. Surgical outcome (Engel classification) was IA in 82% of patients and IC and IID in one patient each, at a median follow‐up period of 2.5 years (range = 1–14.3 years; Table 1, Figure 2B).

No significant differences in seizure outcomes were observed across the various surgical approaches (chi‐squared test, level of significance = .05), with lesionectomies and lobar resections emerging as the most common procedures. In Cohort 1, 67% of patients undergoing lesionectomy and 83% undergoing lobar resection for the last surgery achieved seizure freedom. Similarly, in Cohort 2, all patients who underwent lobar resection and five of six patients who underwent lesionectomy were seizure‐free 1 year postsurgery.

Differences in seizure outcomes were not associated with age at epilepsy onset or surgery, duration from epilepsy onset to surgery, or presurgical seizure frequency (daily, weekly, or monthly; Table S4).

### 3.4 Cognitive outcome

Pre‐ and postsurgical full‐scale IQ score was available in 11 of 25 patients. The median IQ score was 95 (range = 51–116) before and 83 (range = 51–106) after epilepsy surgery. The difference between pre‐ and postsurgical IQ was not statistically significant (Mann–Whitney *U*‐test, *p*‐value = .622, *U*‐value = 68.5).

### 3.5 Safety and functional outcome

In this study, no patient died due to epilepsy surgery or tumor progression in the follow‐up period. Eight of 25 patients experienced additional morbidity after surgery, including homonymous hemianopia, quadrantanopia, hemineglect, facial weakness, hemiparesis (expected in one patient after hemispherotomy), myoclonic seizures, the need for a ventriculoperitoneal shunt, and verbal, learning, and memory deficits (Tables S2 and S3).

## 4 DISCUSSION

Seizures are one of the most common symptoms associated with supratentorial brain tumors, affecting approximately 38% of patients.^28^ Although surgery has proven highly effective in achieving seizure freedom in children with histologically benign, long‐term epilepsy‐associated tumors (LEATs),^20^, ^21^ it is less frequently applied to survivors of high‐grade brain tumors. The reduced expected survival of patients with high‐grade brain tumors may be a key factor in the decision to avoid surgery in these cases. Additionally, the side effects of chemotherapy, such as neutropenia and thrombocytopenia, along with the impacts of CNS irradiation, can increase the risk of surgical complications.^23^

Long‐term survivors of brain cancer often face significant tumor‐ and treatment‐related morbidity, with epilepsy affecting approximately one quarter of all patients and frequently persisting as a chronic symptom in survivors.^4^ Seizures can substantially impair the quality of life and cognitive function of childhood brain cancer survivors.^5^ Furthermore, ASMs in this patient population are associated with notable side effects and may interact with ongoing treatment, such as chemotherapy.^6^ Early consideration of epilepsy surgery in this group can improve both quality of life and cognitive outcomes while allowing for the reduction or cessation of ASM use.^14^, ^16^, ^29^ Cognitive scores before and after epilepsy surgery were available for 11 patients in our study. In contrast to the majority of studies, demonstrating cognitive improvement following epilepsy surgery,^16^, ^17^ these patients showed a nonsignificant decline in mean full‐scale IQ. The timing of the postsurgical cognitive evaluation may have been too early in our study, as cognitive improvement is more likely to be observed with longer follow‐up.^30^ Additionally, the trend was strongly influenced by a single patient, who experienced a significant decline in full‐scale IQ from 116 to 83 (Patient 2, Cohort 1). Nonetheless, the present data are insufficient to draw definitive conclusions about cognitive changes in patients with high‐grade brain tumors undergoing epilepsy surgery. This underscores the need for prospective studies with larger cohorts to better address this question.

This retrospective study demonstrates that epilepsy surgery can be an effective treatment option for children with medically refractory epilepsy and high‐grade CNS tumors. In our study, 80% of the pooled cases achieved seizure freedom, a rate comparable to that in children and adolescents with epilepsies of other etiologies, where 77% were seizure‐free 1 year after surgery.^12^ Specifically, 71% of children previously treated for high‐grade brain tumors attained seizure freedom at the 1‐year follow‐up, whereas 91% of those who initially underwent epilepsy surgery under the assumption of low‐grade tumors achieved seizure freedom. The difference between these groups was not statistically significant, likely due to the small sample size. Also, after a median follow‐up period of 4.3 years following the last epilepsy surgery, an excellent seizure outcome was still observed, with 84% of patients remaining free of disabling seizures (corresponding to Engel class IA–D^24^).

We found that seizure outcomes were not associated with factors such as age at epilepsy onset, age at surgery, duration from epilepsy onset to surgery, or presurgical seizure frequency. In contrast, other studies on epilepsy surgery for LEATs have indicated that a longer duration of epilepsy is linked to poorer seizure outcomes.^31^ We propose that our study may not have detected statistically significant differences due to the limited number of patients.

Contrary to primary tumor surgery, whose main goal is radical resection, epilepsy surgery in our second cohort did not result in any recurrence of cancer following the procedure performed under the initial assumption of low‐grade brain tumors. One patient underwent further surgery upon confirmation of the final histologic diagnosis of a high‐grade brain tumor. Most patients received adjuvant therapies such as chemotherapy or radiation. A possible explanation is the localized nature and early stage of the tumors, as indicated by radiological findings suggestive of low‐grade lesions. Furthermore, LEATs are accompanied by surrounding cortical dysplasia in some cases, also warranting a more extended surgical approach in radiologically suspected low‐grade tumors.^32^

Our data did not show any significant differences in seizure outcomes between lobar resection and lesionectomy, which may be attributed to the small cohort size. Similarly, a prospective study investigating the long‐term outcomes of epilepsy surgery in adults found no significant difference in seizure outcomes between temporal lesionectomies and anterior temporal resections.^33^ The extent of surgery should therefore be determined primarily based on the preoperative assessment, including surface and invasive EEG monitoring and MRI, and should be planned within an interdisciplinary board.

In the first cohort, 45% of patients had either residual tumors, benign lesions, or new malignant tumors in the histological analysis of the resected tissue. These findings raise the possibility that the presence of residual tumor tissue may be an underlying cause for the development of epilepsy, underscoring the need for intensified follow‐up in treated patients with newly emerging seizures. Postradiotherapy lesions can also contribute to the development of seizures. A retrospective study has shown that approximately 5% of pediatric patients with brain tumors develop radiation necrosis after radiotherapy,^34^ which is associated with an increased risk of epilepsy.^35^ Additionally, radiotherapy raises the risk of cortical dysplasia and radiation‐induced gliomas, both of which can contribute to the development of seizures through structural changes.^36^, ^37^

Cohort 2 emphasizes the risk of misdiagnosing brain tumors as low‐grade based on radiological assessments alone, highlighting the necessity for regular radiological monitoring. Additionally, early surgical intervention should be considered, especially when other symptoms, such as drug‐resistant epilepsy, are present.

No patient died due to epilepsy surgery or tumor progression in the follow‐up period. Postsurgical morbidity was observed in 32% of patients and was primarily related to the location and extent of resected brain tissue. This often resulted in expected functional deficits, such as hemianopia or quadrantanopia when surgery involved the visual tract or cortex, and hemiparesis following hemispherotomy. Although this level of postsurgical morbidity may seem high, it aligns with findings from other studies involving extensive surgical approaches.^12^

A limitation of this study is the small cohort size despite drawing on a large European survey, likely due to the rarity of such cases, which limits the generalizability of our findings and the ability to detect significant differences between subgroups. Thus, we failed to detect any significant parameters associated with more favorable seizure outcome. Additionally, due to the retrospective design, the study is subject to certain biases, including selection bias and incomplete data, particularly regarding cognitive outcomes. The short follow‐up period also does not permit conclusions about long‐term seizure freedom. Another limitation is that only nine of 24 tumors were classified using methylation profiling. The integration of molecular diagnostic methods, particularly DNA methylation profiling, has become a critical tool for enhancing the accuracy of brain tumor classification. Histopathological diagnosis without methylation profiling often faces challenges such as interobserver variability, which can result in misdiagnoses or difficulty in reliably classifying certain tumor types.^38^ Studies have shown that approximately 12% of cases are reclassified following DNA methylation analysis.^39^ In one case from our study, a tumor with histological features of a ganglioglioma was initially classified as an anaplastic astrocytoma. However, subsequent analysis of the epilepsy surgery resection revealed components of an anaplastic ganglioglioma. This retrospective finding suggests that the tumor may have been misclassified initially, a situation that could potentially have been avoided with the integration of methylation profiling. Similarly, methylation profiling of a tumor histopathologically classified as a pilocytic astrocytoma tended toward the diagnosis of a pleomorphic xanthoastrocytoma. Analysis of the epilepsy surgery resection performed 4 years later revealed components more consistent with a pleomorphic xanthoastrocytoma, demonstrating that methylation profiling may have provided a more accurate classification of the tumor at that time.

Therefore, it is essential that larger prospective studies be conducted, focusing on cognitive function and quality of life in long‐term survivors of high‐grade brain tumors, particularly in relation to epilepsy. The trade‐off between seizure freedom and neurological or cognitive morbidity is also an important consideration, which would be best evaluated through pre‐ and postsurgical patient/family quality of life surveys.

In conclusion, epilepsy surgery for children with high‐grade brain tumors may yield comparable seizure outcomes to those seen in patients with lower grade tumors, with the majority achieving seizure freedom. Notably, no patients in our study died as a result of epilepsy surgery or progressive tumor disease. Postsurgical morbidity was related to the extent and location of surgery. The total number of only 25 patients despite a European‐wide survey of epilepsy centers suggests that this method is rarely considered in patients with high‐grade tumors and medically refractory epilepsy.

## AUTHOR CONTRIBUTIONS

*Conceptualized the study, conducted data collection, performed statistical analysis, and wrote the manuscript:* Robert Lersch, Till Hartlieb, Ingo Borggraefe, and Christian J. Braun. *Conducted data collection and reviewed the manuscript:* Tom Pieper, Manfred Kudernatsch, Wiebke Hofer, Carmen Barba, Marianna Pommella, Flavio Giordano, Renzo Guerrini, Susanne Schubert‐Bast, Steffen Syrbe, Ricardo Rego, Jorge Pinheiro, Martha Feucht, Roland Coras, Ingmar Blumcke, Alexander Beck, Michael Alber, Moritz Tacke, Jan Rémi, Christian Vollmar, Mathias Kunz, Jay Shetty, Ailsa McLellan, Drahoslav Sokol, Jothy Kandasamy, Kerstin Alexandra Klotz, Victoria San Antonio‐Arce, Andreas Schulze‐Bonhage, Joshua Pepper, William B. Lo, Alexis Arzimanoglou, and Stefano Francione.

## CONFLICT OF INTEREST STATEMENT

None of the authors has any conflict of interest to disclose. We confirm that we have read the Journal's position on issues involved in ethical publication and affirm that this report is consistent with those guidelines.

## Supporting information


Figure S1.

Table S1.

Table S2.

Table S3.

Table S4.

Caption S1.

## Data Availability

All data are included as supplementary files. Additional data may be provided from the corresponding author upon reasonable request.
