# Supplementary figures and images for: Seizure outcomes following epilepsy surgery in pediatric and young adult patients with high‐grade brain tumors: Results from a European survey
Source: Epilepsia. 2025 Mar 1;66(6):1865–75. doi: 10.1111/epi.18323 (PMC12169411; doi:10.1111/epi.18323)

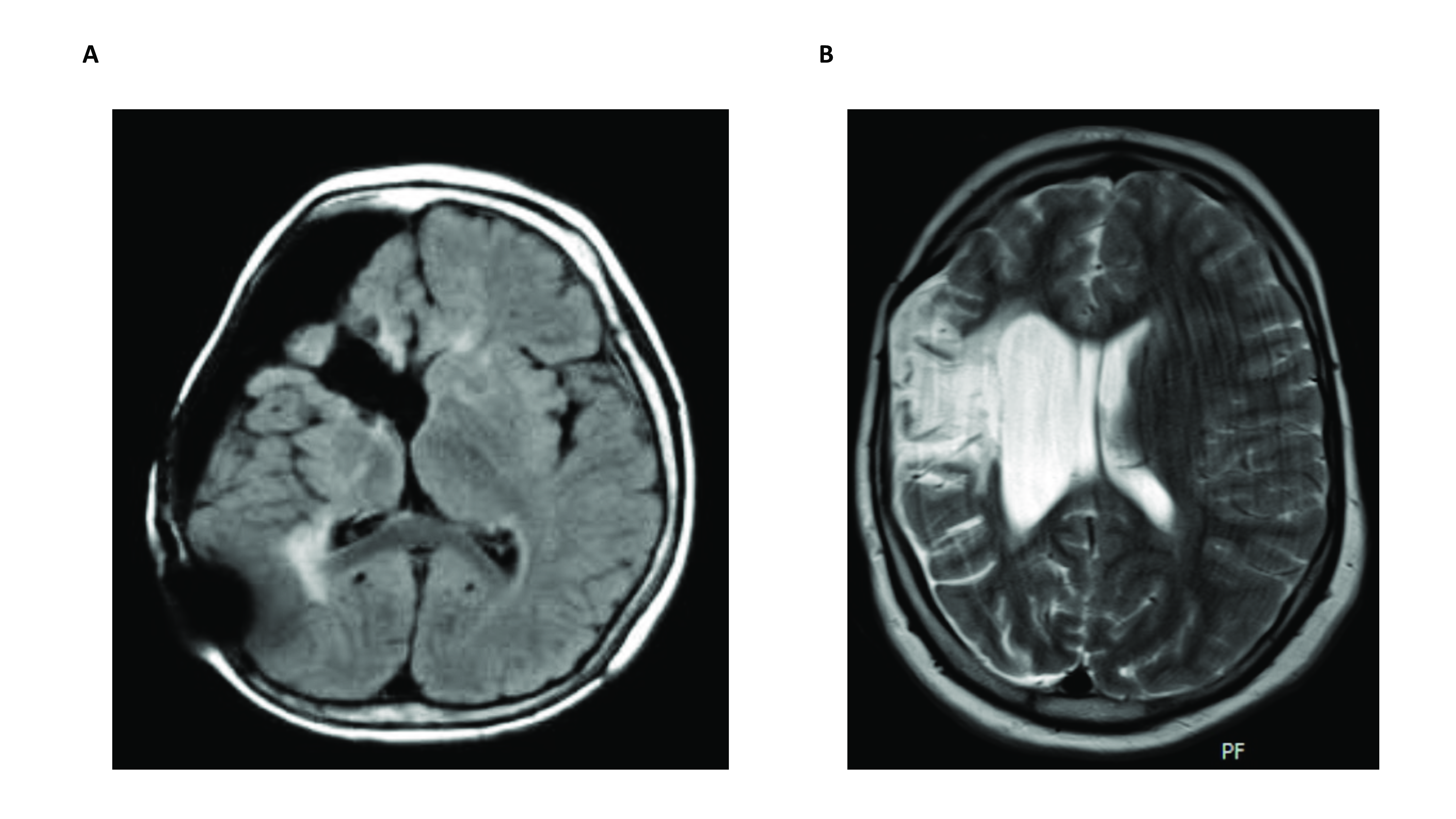

Supplement: Supplementary file 1 — Figure S1. [file EPI-66-1865-s002.tiff]
