# Supplementary material for: Seizure outcomes following epilepsy surgery in pediatric and young adult patients with high‐grade brain tumors: Results from a European survey
Source: Epilepsia. 2025 Mar 1;66(6):1865–75. doi: 10.1111/epi.18323 (PMC12169411; doi:10.1111/epi.18323)
Supplement: Supplementary file 6 — Caption S1. [file EPI-66-1865-s004.docx]

**Table S1: Questionnaire.**

**Table S2:** **Individual patient data cohort (i).**

**Table S3:** **Individual patient data cohort (ii).**

**Table S4: Seizure outcome analysis.**

**Figure S1: Preoperative MRI sections of two patients who underwent hemispherotomy for epilepsy surgery. A,** Patient 4 cohort (i), FLAIR. **B,** Patient 5 cohort (i), T2-weighted.
